# Psychometric properties of the health-promoting lifestyle profile II: cross-cultural validation of the Malay language version

**DOI:** 10.1186/s12889-019-7109-2

**Published:** 2019-06-13

**Authors:** Garry Kuan, Yee Cheng Kueh, Nurzulaikha Abdullah, Evelyn Li Min Tai

**Affiliations:** 1 Exercise and Sports Science Programme, School of Health Sciences, Universiti Sains Malaysia, Kubang Kerian, Kelantan Malaysia; 2 Unit of Biostatistics and Research Methodology, School of Medical Sciences, Universiti Sains Malaysia, Kubang Kerian, Kelantan Malaysia; 3 Department of Ophthalmology, School of Medical Sciences, Health Campus, Universiti Sains Malaysia, Kubang Kerian, Kelantan Malaysia; 4 Hospital Universiti Sains Malaysia, Kubang Kerian, Kelantan Malaysia

**Keywords:** Healthy lifestyles, Health behaviour, Confirmatory factor analysis, Reliability, Validity, Latent variable

## Abstract

**Background:**

Health-promoting behaviour is an important concept for health education. Unfortunately, there is a dearth of validated instruments to measure levels of health-promoting behaviour in the Malaysian context. The purpose of this study was to validate a Malay-language version of the Health-Promoting Lifestyle Profile II (HPLP-II) using a confirmatory approach.

**Methods:**

Participants were 997 university undergraduate students, with a mean age of 21 years (SD = 1.58). The majority of the participants (80.4%) were female. Health-promoting behaviour was assessed using the 52-item HPLP-II, which measures six components of health-promoting behaviour outcomes. HPLP-II was translated into the Malay language using standard forward and backward translation procedures. Participants then completed the HPLP-II Malay version (HPLP-II-M). Confirmatory factor analysis (CFA) was conducted using Mplus 8.0 software on the six domains of HPLP-II-M model.

**Results:**

The CFA result based on the hypothesised measurement model of six factors was aligned with the original HPLP-II, except for two low loading items which were subsequently removed from the CFA analysis. The final CFA measurement model with 50 items resulted in a good fit to the data based on RMSEA and SRMR fit indices (RMSEA = 0.046, 90%CI = 0.045, 0.048, SRMR = 0.062). The construct reliabilities for the HPLP-II-M subscales were acceptable, ranging from 0.737 to 0.878.

**Conclusion:**

The HPLP-II-M with six components of health-promoting behaviour outcomes and 50 items was considered valid and reliable for the present Malaysian sample.

**Electronic supplementary material:**

The online version of this article (10.1186/s12889-019-7109-2) contains supplementary material, which is available to authorized users.

## Background

The global increase in the prevalence of non-communicable diseases is a public health problem which has the potential to overwhelm healthcare systems worldwide [1–3]. Health promotion, as reflected in a healthy lifestyle, is an integral part of disease prevention [4, 5]. Health-promoting behaviour has been associated not only with improved physical and mental health outcomes [6–9], but also with lower healthcare costs [10].

Evaluation of health-promoting behaviour is essential in primary care research to provide data for preventive interventions, facilitate policy-making and empower patients’ self-management of their health behaviours. Such research necessitates the use of reliable and valid measurement tools, which serve to enhance the international comparability of health data. The Health-Promoting Lifestyle Profile II (HPLP-II) is a widely-used instrument for evaluation of health behaviour [11, 12] which has been validated in multiple studies [13–15]. Based on Pender’s Health Promotion Model, it conceptualises an individual’s health-promoting lifestyle in terms of the following dimensions; health responsibility, physical activity, nutrition, spiritual growth, interpersonal relations and stress management.

The HPLP-II has been translated and psychometrically validated across several linguistic and cultural groups. The translated versions of HPLP-II include Portuguese [14], Chinese [16, 17], Spanish [18], Iranian [19], Japanese [20], Turkish [21], Italian [22] and Arabic [23]. Several abbreviated versions of HPLP-II have emerged from these studies, with the total number of items ranging from 26 to 51 items [16–19, 22]. These studies found the translated versions of HPLP-II to be valid and reliable. The majority of studies reported the validation results based on exploratory factor analysis and/or confirmatory factor analysis and internal consistency reliability based on Cronbach’s alpha coefficient.

The Malay language, which is the primary language of the Austronesian family and the official language of Malaysia, is spoken as a native language by more than 200 million people in South-East Asia [24, 25]. A Malay translation of the HPLP-II is essential for communities in which Malay is the predominant language spoken by that population. In places where Malay is the only medium of communication, it is imperative that healthcare providers use the language of the people. This is especially the case in rural areas, where underprivileged and low literacy populations are in dire need of health education [26–28].

Testing of validity and reliability of translated instruments is necessary because adaptation of a health behaviour instrument to different populations requires not only language, but linguistic and cultural adjustment [29]. Up to the present date, only the English version of HPLP-II has been validated among Malaysian populations [30]. Furthermore, the authors of that study only focused on three out of six subscales (i.e., health responsibility, nutrition and physical activity). Since Malay is the main language spoken by Malaysian populations and the previous validation study did not include the full 52 items of HPLP-II, there is a pressing need for a validated full version of the HPLP-II in Malay language. Our study aimed to translate the English version of HPLP-II into the Malay language and examine its psychometric properties among Malaysian population.

## Methods

### Participants

A total of 997 university undergraduate students in Universiti Sains Malaysia participated in this study. The majority were female (80.4%). The mean age of the participants was 21 years old (SD = 1.58). Most of the participants were ethnic Malays (77.5%).

### Measures

The HPLP-II focuses on self-initiated actions and perceptions that serve to maintain or enhance the level of wellness, self-actualisation and fulfillment of the individual [31]. The HPLP-II has been translated into different languages and widely used in other studies [14, 16, 19, 32]. The internal consistency Cronbach alpha for the original English version of HPLP-II was satisfactory, with 0.94 for the total scale of HPLP-II, and from 0.79 to 0.87 for its six subscales [31].

The Health-Promoting Lifestyle Profile II – Malay language (HPLP-II-M) is the translated version of the HPLP-II [30], consisting of 52 items that measure health-promoting behaviour or habits. It contains six domains or factors, namely health responsibility (nine items), physical activity (eight items), nutrition (nine items), spiritual growth (nine items), interpersonal relations (nine items), and stress management (eight items). It uses a four-point response scale that indicates respondents’ frequency of engaging in each behavior. Items are scored as 1 = “*never*”, 2 = “*sometime*”, 3 = “*often*”, 4 = “*routinely*”.

### Questionnaire translation

Permission to use and translate the HPLP-II into Malay language was obtained from the main author. The original English language version of HPLP-II was translated into Malay language using the standard forward and backward translation procedure recommended by Brislin [33]. First, one of the bilingual authors familiar with the content did the forward translation (English to Malay), followed by a backward translation (Malay to English) by another bilingual expert. Next, a panel of five experts competent in both languages (consisting of a health psychologist, sport psychologist, clinician, experienced linguist and psychometric expert) reviewed the Malay translation from English, and English translation from Malay, comparing each item to the corresponding item on the original English version. The experts were also asked to check the content of HPLP-II-M to ensure that the items were culturally appropriate to the Malaysian population. The final version of HPLP-II-M was pre-tested among 10 university undergraduate students for clarity, comprehension and understanding. The students answered the HPLP-II-M questionnaire and were asked to comment on the wording and the presentation of the questionnaire. The result of the pre-test among the students was good and no modifications were necessary. A copy of the questionnaire (HPLP-II-M) is available in the Additional file 1.

### Data collection

A cross-sectional study design was employed on the self-administered HPLP-II-M questionnaire. Convenience sampling method was used for recruitment of participants. Data collection was performed between October 2016 to January 2017 at Universiti Sains Malaysia, Kelantan, Malaysia. The study was approved by the Universiti Sains Malaysia Human Research Ethics Committee and conducted in accordance with the Declaration of Helsinki.

Malaysian undergraduate students who comprehended the Malay language and were willing to complete the questionnaire during the data collection period were included in the study. Lectures which comprised of medical, dental and health sciences undergraduate students were identified. Students were briefed regarding the study at the end of their lectures. Study information sheets, the HPLP-II-M and a socio-demographic survey form were distributed to the students. The form contained several questions related to aspects of health behaviour, including age, gender, ethnicity, smoking status, presence of medical illness, physical activities and frequency per week of pursuing these activities.

Participants who volunteered to participate in the study completed the demographic sheet and the HPLP-II-M and returned it to the researcher. Participants’ responses were non-identifiable because the questionnaires were anonymous. Implied consent was obtained when participants completed and returned the questionnaire to the researchers. No incentives were given to the participants for the study. A total of 1100 questionnaires were distributed to the eligible participants and 997 completed questionnaires were returned to the researchers. The response rate was 91%.

### Statistical analysis

Statistical analyses were conducted using Mplus 8.0 software. Frequency analyses were used to identify missing values in each variable. All questionnaires included in the study were completed without any missing values. Multivariate normality assumption was checked, and results indicated that the data did not meet the assumption, based on Mardia multivariate skew (*p* < 0.001) and kurtosis (*p* < 0.001) tests. Therefore, for the subsequent confirmatory factor analysis (CFA), the robust maximum likelihood estimator (MLR) was utilized, as this is robust to non-normality. To achieve good psychometric characteristics, high standardised factor loadings (> 0.40) are preferred [34]. Therefore, items with factor loadings less than 0.40 were examined and treated as potentially problematic items. Problematic items would be omitted only with adequate theoretical support. According to Hair et al., reporting various fit indices is necessary because there are no standard rules for assessment of model fit [35]. Based on the 6-factor structure and 52-item measurement model in the present study, the fit indices and its acceptable threshold value are as follows: the comparative fit index (CFI) and Tucker and Lewis index (TLI) with a desired value of more than 0.90; the root mean square error of approximation (RMSEA) with a desired value of less than 0.08; probability RMSEA with the desired value of more than 0.05; and the standardised root mean square (SRMR) with a desired value of less than 0.08 [35]. The construct reliability (CR) in a latent variable modeling approach was calculated for each factor in HPLP-II-M based on CR formula listed in a published study by Raykov and Marcoulides in 2015 [36]. The acceptable value of CR is above 0.70 [35]. Discriminant validity was checked by inspecting the correlation between the factors in the model. Discriminant validity is established when the correlation between factors is below 0.85.

## Results

### Characteristics of participants

On average, the participants engaged in physical activities twice a week, with each session lasting 30 min. The majority of participants did not have any illnesses and were non-smokers. Other descriptive statistics are presented in Table 1.

**Table 1:** Participants’ characteristics (*n* = 997)

Variables	Mean (SD)	n (%)
Age	20.5 (1.58)	
Gender		
Male		195 (19.6)
Female		802 (80.4)
Ethnicity		
Malay		773 (77.5)
Chinese		135 (13.5)
Indian		57 (5.7)
Others		32 (3.2)
Undergraduate course		
Medical Sciences		282 (28.3)
Nursing		227 (22.8)
Biomedical sciences		105 (10.5)
Dental sciences		89 (8.9)
Forensic sciences		64 (6.4)
Occupational health		63 (6.3)
Nutrition sciences		60 (6.0)
Sport sciences		54 (5.4)
Dietetic		53 (5.3)
Physical activity		
Yes		846 (84.9%)
No		151 (15.1)
Frequency session (per week)	2.1 (1.57)	
Duration in minute (per session)	51.5 (41.72)	
Illness:		
Yes		38 (3.8)
No		959 (96.2)
Consume medication		
Yes		22 (2.2)
No		975 (97.8)
Smoker		
Yes		45 (4.5)
No		952 (95.5)

### Descriptive statistics of the HPLP-II-M

Table 2 presents the frequency and percentage of the respondents for each item in the HPLP-II-M based on a 5-point Likert scale. The mean and standard deviation (SD) for each item in the HPLP-II-M are also presented.

**Table 2:** Descriptive statistics of items for the HPLP-II-M

Item	Four rating likert scale, n (%)				Mean (SD) items	Mean (SD) subscales
	Never	Sometime	Often	Routinely		
HR:						2.39(0.62)
Q3	137(13.7)	465(46.6)	299(30.0)	96(9.6)	2.36(0.84)	
Q9	67(6.7)	443(44.4)	364(36.5)	123(12.3)	2.54(0.79)	
Q15	217(21.8)	390(39.1)	280(28.1)	110(11.0)	2.28(0.93)	
Q21	155(15.5)	359(36.0)	375(37.6)	108(10.8)	2.44(0.88)	
Q27	197(19.8)	407(40.8)	273(27.4)	120(12.0)	2.32(0.92)	
Q33	158(15.8)	385(38.6)	326(32.7)	128(12.8)	2.43(0.91)	
Q39	229(23.0)	378(37.9)	279(28.0)	111(11.1)	2.99(0.70)	
Q45	154(15.4)	449(45.0)	276(27.7)	118(11.8)	2.87(0.70)	
Q51	105(10.5)	372(37.2)	383(38.4)	137(13.7)	2.55(0.86)	
PA:						2.58(0.53)
Q4	107(10.7)	448(44.9)	328(32.9)	114(11.4)	2.45(0.83)	
Q10	39(3.9)	412(41.3)	371(37.2)	175(17.6)	2.68(0.80)	
Q16	59(5.9)	376(37.7)	399(40.00	163(16.3)	2.67(0.82)	
Q22	46(4.6)	407(40.8)	390(39.1)	154(15.4)	2.65(0.79)	
Q28	62(6.2)	386(8.7)	410(41.1)	139(13.9)	2.63(0.80)	
Q34	28(2.8)	263(26.4)	527(52.9)	179(18.0)	2.81(0.73)	
Q40	169(17.0)	401(40.2)	324(32.5)	103(10.3)	2.70(0.81)	
Q46	177(17.8)	382(38.3)	328(32.9)	110(11.0)	2.92(0.76)	
N:						2.57(0.50)
Q2	46(4.6)	481(48.2)	341(34.2)	129(12.9)	2.55(0.77)	
Q8	27(2.7)	418(41.9)	386(38.7)	166(16.6)	2.69(0.78)	
Q14	177(17.8)	439(44.0)	264(26.5)	117(11.7)	2.32(0.90)	
Q20	63(6.3)	517(51.9)	297(29.8)	120(12.0)	2.48(0.79)	
Q26	66(6.6)	395(39.6)	379(38.0)	157(15.7)	2.63(0.83)	
Q32	133(13.3)	470(47.1)	281(28.2)	113(11.3)	2.38(0.85)	
Q38	28(2.8)	308(30.9)	504(50.6)	157(15.7)	3.01(0.69)	
Q44	54(5.4)	360(36.1)	412(41.3)	171(17.2)	2.63(0.88)	
Q50	15(1.5)	287(28.8)	457(45.8)	238(23.9)	2.92(0.76)	
SG:						2.94(0.45)
Q6	19(1.9)	256(25.7)	521(52.3)	201(20.2)	2.86(0.73)	
Q12	10(1.0)	130(13.0)	544(54.6)	313(31.4)	3.16(0.68)	
Q18	8(0.8)	163(16.3)	591(59.3)	235(23.6)	3.06(0.66)	
Q24	21(2.1)	272(27.3)	536(53.8)	168(16.9)	2.85(0.71)	
Q30	6(0.6)	176(17.7)	576(57.8)	239(24.0)	3.05(0.66)	
Q36	24(2.4)	279(28.0)	524(52.6)	170(17.1)	2.36(0.88)	
Q42	12(1.2)	193(19.4)	563(56.5)	229(23.0)	2.37(0.90)	
Q48	108(10.8)	314(31.5)	417(41.8)	158(15.8)	2.94(0.73)	
Q52	12(1.2)	256(25.7)	506(50.8)	223(22.4)	2.94(0.73)	
IR:						2.84(0.44)
Q1	36(3.6)	422(42.3)	432(43.3)	107(10.7)	2.61(0.72)	
Q7	30(3.0)	223(22.4)	546(54.8)	198(19.9)	2.91(0.73)	
Q13	14(1.4)	177(17.8)	574(57.6)	232(23.3)	3.03(0.68)	
Q19	21(2.1)	271(27.2)	523(52.5)	182(18.3)	2.87(0.72)	
Q25	11(1.1)	265(26.6)	530(53.2)	191(19.2)	2.90(0.70)	
Q31	43(4.3)	268(26.9)	497(49.8)	189(19.0)	2.83(0.78)	
Q37	214(21.5)	337(33.8)	338(33.9)	108(10.8)	2.43(0.91)	
Q43	17(1.7)	201(20.2)	557(55.9)	222(22.3)	2.84(0.72)	
Q49	15(1.5)	274(27.5)	538(54.0)	170(17.1)	2.87(0.70)	
SM:						2.81(0.44)
Q5	28(2.8)	331(33.2)	456(45.7)	182(18.3)	2.79(0.77)	
Q11	8(0.8)	191(19.2)	578(58.0)	220(22.1)	3.01(0.67)	
Q17	40(4.0)	345(34.6)	456(45.7)	156(15.6)	2.73(0.77)	
Q23	14(1.4)	239(24.0)	581(58.3)	163(16.3)	2.90(0.67)	
Q29	7(0.7)	215(21.6)	581(58.3)	194(19.5)	2.96(0.66)	
Q35	20(2.0)	319(32.0)	500(50.2)	158(15.8)	2.27(0.94)	
Q41	174(17.5)	341(34.2)	362(36.3)	120(12.0)	2.36(0.88)	
Q47	27(2.7)	266(26.7)	541(54.3)	163(16.3)	2.55(0.86)	

*HR* health responsible, *PA* physical activity, *N* nutrition, *SG* spiritual growth, *IR* interpersonal relations, *SM* stress management

### Measurement models of HPLP-II-M

Results of models tested with fit indices are summarised in Table 3. The hypothesised measurement model for HPLP-II-M consisted of six factors with 52 items. The number of items within each factor varied. The hypothesised measurement model fit the data well based on RMSEA and SRMR fit indices. Factor loadings ranged from 0.339 to 0.777 (see Table 4). There were two items with factor loading less than the acceptable value of 0.40. Item Q1 (*Discuss my problems and concerns with people close to me*), which had the lowest factor loading (0.339), was removed and the fit indices were re-examined (RMSEA = 0.045, 90%CI = 0.045, 0.048, CIfit = 1.000, SRMR = 0.062, CFI = 0.816, TLI = 0.806). The fit indices did not improve substantially. The CFI and TLI were below the threshold value of 0.90. The next item removed was Q50 (*Eat breakfast*), which had the factor loading of 0.373 after removing Q1. The fit indices of CFI and TLI were still below the threshold value of 0.90. The fit indices of the measurement model after removal of the two items are presented in Table 3 (Model 2). No further model modifications were considered important and theoretically appropriate in improving the fit indices, especially the CFI and TLI values, based on modification indices and residual variance between items.

**Table 3:** Summary of fit indices of measurement models for HPLP-II-M

Model	RMSEA (90% CI)	Probability RMSEA	SRMR	CFI	TLI
Model 1	0.046 (0.044,0.048)	1.000	0.062	0.814	0.805
Model 2	0.046 (0.045,0.048)	1.000	0.062	0.821	0.811

*CI*, Confidence interval

**Table 4:** Standardised factor loadings and construct reliabilities for measurement model 1 and 2 of HPLP-II-M

Construct and Items	Standardized factor loadings (*λ* ^a^)	Standardized factor loadings (*λ* ^b^)	Composite Reliability (CR^a^)	Composite Reliability (CR^b^)
HR				
Q3	0.601	0.601	0.874	0.878
Q9	0.477	0.477		
Q15	0.749	0.749		
Q21	0.663	0.662		
Q27	0.755	0.756		
Q33	0.578	0.578		
Q39	0.777	0.778		
Q45	0.711	0.771		
Q51	0.593	0.591		
PA				
Q4	0.582	0.582	0.805	0.805
Q10	0.555	0.553		
Q16	0.577	0.576		
Q22	0.600	0.600		
Q28	0.620	0.620		
Q34	0.458	0.457		
Q40	0.628	0.629		
Q46	0.642	0.642		
N				
Q2	0.552	0.551	0.777	0.774
Q8	0.479	0.474		
Q14	0.542	0.545		
Q20	0.585	0.595		
Q26	0.578	0.576		
Q32	0.604	0.612		
Q38	0.458	0.456		
Q44	0.575	0.566		
Q50	0.373	–		
SG				
Q6	0.532	0.529	0.811	0.811
Q12	0.556	0.557		
Q18	0.589	0.589		
Q24	0.581	0.581		
Q30	0.635	0.636		
Q36	0.570	0.571		
Q42	0.600	0.601		
Q48	0.486	0.485		
Q52	0.562	0.562		
IR				
Q1	0.339	–	0.742	0.737
Q7	0.429	0.425		
Q13	0.534	0.536		
Q19	0.459	0.453		
Q25	0.587	0.589		
Q31	0.500	0.497		
Q37	0.426	0.421		
Q43	0.587	0.588		
Q49	0.555	0.555		
SM				
Q5	0.403	0.404	0.744	0.746
Q11	0.428	0.430		
Q17	0.486	0.486		
Q23	0.532	0.534		
Q29	0.592	0.593		
Q35	0.604	0.605		
Q41	0.498	0.502		
Q47	0.579	0.578		

Note. ^a^ Model 1, ^b^ Model 2, *Q* item number in HPLP-II-M, *HR* health responsible, *PA* physical activity, *N* nutrition, *SG* spiritual growth, *IR* interpersonal relations, *SM* stress management

The CR values for Model 1 ranged from 0.742 to 0.874, while that of Model 2 ranged from 0.737 to 0.878 (Table 4). The CR values did not improve the factors ‘interpersonal relations’ and ‘nutrition’ after removing the two low loading items (i.e., Q1 and Q50) in the measurement model. However, the reliabilities for all factors in Model 1 and Model 2 were considered satisfactory. The final measurement model of HPLP-II-M (Model 2) consisted of all 50 items and six factors in the present confirmatory study with removal of two items (i.e., Q1 and Q50) due to low factor loading.

### Discriminant validity

The discriminant validity was checked based on the correlations among the factors. Table 5 presents the correlation value and its significant indication for Model 1. All the correlations were below the recommended cut-off point of 0.85, which indicated that the six health-promoting behaviour factors achieved good discriminant validity.

**Table 5:** Correlations between latent variables in Model 1 for HPLP-II-M

Variable	Health Responsibility	Physical Activity	Nutrition	Spiritual Growth	Interpersonal Relations	Stress Management
1. Health Responsibility	1	0.70^a^	0.71^a^	0.43^a^	0.55^a^	0.55^a^
2. Physical Activity		1	0.68^a^	0.53^a^	0.54^a^	0.65^a^
3. Nutrition			1	0.48^a^	0.54^a^	0.57^a^
4. Spiritual Growth				1	0.73^a^	0.71^a^
5. Interpersonal Relations					1	0.67^a^
6. Stress Management						1

^a^Correlation is significant at the 0.05 level (two tailed)

## Discussion

The development of the HPLP-II-M and evaluation of its validity and reliability is a vital step in the determination of health-promoting behaviour among the Malay-speaking population. The original English version of HPLP-II has been shown to be reliable, valid and stable across time based on previous studies [18, 37]. It has been widely translated and used to measure health-promoting behaviour in different study populations [16, 17, 38–43]. Although Malays comprise a significant proportion of the South East Asian population, there is a severe lack of validated tools in the Malay language to measure health-promoting behaviour. Our translation of the English HPLP-II into Malay (HPLP-II-M) fills this gap and will serve as a useful tool in health education, clinical management and research in populations where Malay is the lingua franca.

This study applied CFA, which is a type of structural equation modelling that deals specifically with measurement models. CFA examines the strength of the relationship between an observed measure and latent variables or factors, based on factor loading. When dealing with CFA, the number of factors is specified. CFA requires a strong conceptual foundation to guide the specification and evaluation of the factor model. It can be utilised in psychometric evaluation, detection of method effects, construct validation and also for the evaluation of measurement in variance. Since the factors and items of HPLP-II have been predetermined in previous studies, we conducted only a confirmatory study on the translated version of HPLP-II-M. In the confirmatory study, our aim was to confirm if the six-factor 52 item measurement model fit the data well. Our results confirm the validity and reliability of HPLP-II-M with 50 items, based on the CFA analysis. Furthermore, the discriminant validity of the factors in HPLP-II-M was found to be satisfactory, indicating that the latent variables in the CFA measurement model of HPLP-II-M contribute information which is not captured by the other latent variables.

Previous validation studies of HPLP-II reported varying total number of items which fit the CFA measurement model. For example, based on psychometric properties, Pérez-Fortis et al. retained 44 items for Spanish version [18], Teng et al., Meihan and Chung-Ngok retained 30 items and 51 items respectively for the Chinese version [16, 17], while Savarese et al. retained 26 items for the Italian version of the HPLP-II [22]. The present study retained 50 items in the final measurement model, after removal of two low factor loading items. Our CFA analysis of HPLP-II-M supported the six factor structure proposed by the authors of the original HPLP-II. These results are consistent with other studies of the psychometric properties of the HPLP-II in Chinese and Spanish samples [16, 18].

The two items (Q1 [interpersonal relations]: *Discuss my problems and concerns with people close to me*; Q50 [nutrition]: *Eat breakfast*) removed in the present study had factor loading lower than 0.40, which is the lowest threshold we identified in the present study. These items also were found to be problematic in other validation studies. Item Q1 was removed in the Chinese validation study during EFA stage [17], and item Q50 was removed in the Spanish study due to very low corrected item-total correlation value < 0.20 [18]. Item Q50 was also removed in the Italian study due to its contribution to low reliability of the scale [22] and Chinese validation study due to its insignificant factor loadings in a CFA analysis [16]. Removal of these items improved the fit of the model in those studies as well as in the present study.

The majority of previous studies reported the reliability of HPLP-II-M based on Cronbach alpha. The present study reported reliability based on the CR formula presented by Raykov and Marcoulides in 2015 [36]. CR has been recommended as a reliability test for CFA measurement model instead of Cronbach’s alpha [44]. This is due to the fact that Cronbach’s alpha can over- or underestimate scale reliability at the population level [45]. In the present study, the reliability value based on CR for all subscales of HPLP-II-M was satisfactory and above the recommended value of 0.70 [35]. In order to compare the reliability of HPLP-II-M with other published studies on HPLP-II, we computed the reliability of HPLP-II-M with 50 items based on Cronbach alpha. The reliability was satisfactory, with Cronbach alpha of 0.94 for overall scale, 0.87 for health responsibility, 0.81 for physical activity, 0.77 for nutrition, 0.81 for spiritual growth, 0.73 for interpersonal relations, and 0.74 for stress management. These results were consistent with the study conducted using the original English version of HPLP-II with 52 items. The overall reliability scale was reported as 0.94 and reliability for six subscales ranged from 0.79 to 0.87 [37].

The HPLP-II-M is an easily-administered instrument for evaluation of effectiveness of public health interventions on health-promoting behaviour. Strengths of our study include its large sample size and its multi-racial population of Malay-speaking participants. Few limitations of this study must be acknowledged. First, this was a cross-sectional study, which precludes the inference of any temporal relations. Longitudinal studies to examine the change and stability of HLPL-II-M across time will consolidate our findings [46]. A second limitation is the use of a self-reported questionnaire, which may be subject to response bias. In order to overcome this limitation, we emphasised the importance of honest feedback to the subjects prior to data collection. Given that the questionnaires were answered anonymously, the chances of participants responding in a socially acceptable manner were reduced. A third limitation is that the data consisted of mainly female students. This reflects the gender enrolment ratio of Malaysia’s public universities for the past two decades, in which females comprise almost two-thirds of the student population [47, 48]. Other previous studies in our university also observed a high proportion of female respondents, ranging from 63 to 81% [49–52]. To obtain an equal ratio of male and female respondents, future studies should utilize stratified sampling method rather than convenience sampling method. Finally, the study participants were undergraduate students, with ages ranging from 18 to 31 years old. Although an understanding of health-promoting behavior among young adults is important as it influences health choices in later life [53], further research should examine the replicability of the HPLP-II-M in more diverse Malay-speaking populations of varying ages, education levels, occupations and health.

## Conclusion

The final measurement model for the HPLP-II-M questionnaire in the present sample consisted of 50 items and six subscales. Two items with low factor loading items were removed in the present confirmatory study. Future research on health-promoting behaviour among populations where Malay is the major language can utilize the HPLP-II-M, interpreting their responses within the six-factor framework of subscales.

## Additional file

Additional file 1: The Malay version of the Health-Promoting Lifestyle Profile II. (PDF 60 kb)

## Data Availability

Please contact corresponding author for dataset requests.

## Abbreviations

CFA: Confirmatory factor analysis
CFI: Comparative fit index
CR: Construct reliability
HPLP-II: Health-Promoting Lifestyle Profile II
HPLP-II-M: Malay version of the Health-Promoting Lifestyle Profile II
MLR: Robust maximum likelihood estimator
RMSEA: Root mean square error of approximation
SD: Standard deviation
SRMR: Standardised root mean square
TLI: Tucker-lewis index
