# Supplementary material for: Psychometric properties of the health-promoting lifestyle profile II: cross-cultural validation of the Malay language version
Source: BMC Public Health. 2019 Jun 13;19:751. doi: 10.1186/s12889-019-7109-2 (PMC6567916; doi:10.1186/s12889-019-7109-2)
Supplement: Supplementary file 1 — The Malay version of the Health-Promoting Lifestyle Profile II. (PDF 60 kb) [file 12889_2019_7109_MOESM1_ESM.pdf]

Profil Galakan-Gaya Hidup Sihat II

Soal selidik ini mengandungi pernyataan tentang cara hidup semasa atau tabiat peribadi anda. Sila berikan respon untuk setiap pernyataan secara tepat, dan cuba menjawab semua pernyataan. Nyatakan kekerapan yang anda lakukan dalam setiap tingkah laku dengan membulatkan skala yang sesuai.

	Tidak pernah	Kadang-kadang	Selalu	Secara berkala
1. Membincangkan masalah dan kebimbangan saya dengan orang yang rapat dengan saya.	T	K	S	B
2. Memilih diet yang rendah lemak, lemak tepu dan kolestrol.	T	K	S	B
3. Melaporkan sebarang tanda atau gejala yang ganjil kepada doktor atau pakar kesihatan yang lain.	T	K	S	B
4. Mengikuti program senaman yang telah dirancang.	T	K	S	B
5. Mendapatkan tidur yang cukup.	T	K	S	B
6. Merasakan saya membesar dan berubah dalam cara yang positif.	T	K	S	B
7. Dengan mudahnya saya memuji orang lain atas pencapaian mereka.	T	K	S	B
8. Menghadkan pengambilan gula dan makanan yang mengandungi gula (manis).	T	K	S	B
9. Membaca atau menonton program TV mengenai peningkatan kesihatan.	T	K	S	B
10. Bersenam bersungguh-sungguh selama 20 minit atau lebih sekurang-kurangnya tiga kali seminggu (seperti berjalan pantas, berbasikal, menari areobik, menggunakan tangga pendaki).	T	K	S	B
11. Mengambil sedikit masa untuk berehat setiap hari.	T	K	S	B
12. Percaya bahawa hidup saya mempunyai tujuan.	T	K	S	B
13. Mengekalkan hubungan yang bermakna dan memuaskan dengan orang lain.	T	K	S	B
14. Makan 6-11 hidangan roti, bijirin, nasi dan pasta setiap hari.	T	K	S	B
15. Bertanya pada pakar kesihatan untuk memahami arahan mereka.	T	K	S	B
16. Mengambil bahagian dalam aktiviti fizikal pada tahap ringan ke sederhana (seperti berjalan secara berterusan selama 30-40 minit 5 kali atau lebih setiap minggu).	T	K	S	B

17. Menerima perkara yang saya tidak boleh ubah dalam kehidupan saya.	T	K	S	B
18. Melihat ke masa hadapan.	T	K	S	B
19. Menghabiskan masa dengan kawan rapat.	T	K	S	B
20. Makan 2-4 hidangan buah setiap hari.	T	K	S	B
21. Mendapatkan pandangan kedua apabila saya mengesyaki nasihat yang diberi oleh pakar kesihatan saya.	T	K	S	B
22. Mengambil bahagian dalam aktiviti fizikal masa lapang (rekreasi) (seperti berenang, menari, dan berbasikal).	T	K	S	B
23. Menumpukan perhatian kepada pemikiran yang menyenangkan sebelum tidur.	T	K	S	B
24. Berasa puas dan tenang dengan diri sendiri.	T	K	S	B
25. Mendapati bahawa adalah mudah untuk menunjukkan keprihatinan, kasih-sayang dan kemesraan kepada orang lain.	T	K	S	B
26. Makan 3-5 hidangan sayur-sayuran setiap hari.	T	K	S	B
27. Berbincang mengenai kebimbangan terhadap kesihatan saya dengan pakar kesihatan.	T	K	S	B
28. Melakukan senaman regangan sekurang-kurangnya 3 kali seminggu.	T	K	S	B
29. Menggunakan cara tertentu untuk mengawal tekanan saya.	T	K	S	B
30. Berusaha ke arah matlamat jangka masa panjang dalam hidup saya.	T	K	S	B
31. Menyentuh dan tersentuh oleh orang yang saya hargai.	T	K	S	B
32. Makan 2-3 hidangan susu, yogurt atau keju setiap hari.	T	K	S	B
33. Memeriksa badan saya sekurang-kurangnya sebulan sekali untuk mengesan sebarang perubahan fizikal atau tanda berbahaya.	T	K	S	B
34. Bersenam semasa melakukan aktiviti-aktiviti harian (seperti berjalan semasa makan tengahari, menggunakan tangga berbanding lif, meletakkan kereta jauh dari destinasi dan berjalan).	T	K	S	B
35. Menyeimbangkan masa antara bekerja dan bermain.	T	K	S	B
36. Mendapati bahawa setiap hari adalah menarik dan mencabar.	T	K	S	B

37. Mencari jalan untuk memenuhi kehendak intim saya.	T	K	S	B
38. Makan hanya 2-3 hidangan berasaskan daging, ayam, ikan, kacang kering, telur dan kumpulan kacang setiap hari.	T	K	S	B
39. Bertanyakan maklumat dari pakar kesihatan mengenai cara untuk menjaga diri saya.	T	K	S	B
40. Memeriksa kadar denyutan nadi saya semasa bersenam.	T	K	S	B
41. Mengamalkan bersantai atau meditasi selama 15-20 minit setiap hari.	T	K	S	B
42. Tahu tentang apa yang penting dalam hidup saya.	T	K	S	B
43. Mendapatkan sokongan daripada rangkaian orang yang penyayang.	T	K	S	B
44. Membaca label untuk mengenalpasti nutrien, lemak, kandungan sodium dalam makanan yang berbungkus.	T	K	S	B
45. Menghadiri program pendidikan mengenai penjagaan kesihatan peribadi.	T	K	S	B
46. Mencapai sasaran kadar degupan jantung saya semasa bersenam.	T	K	S	B
47. Menenangkan diri saya untuk mengelakkan keletihan.	T	K	S	B
48. Berasa berhubung dengan kuasa yang lebih besar daripada diri saya.	T	K	S	B
49. Menyelesaikan masalah dengan orang lain melalui perbincangan dan persepakatan.	T	K	S	B
50. Makan sarapan.	T	K	S	B
51. Mencari panduan dan kaunseling bila diperlukan.	T	K	S	B
52. Mendedahkan diri saya dengan pengalaman baru dan cabaran.	T	K	S	B
